# Developmental Dyslexia: Environment Matters

**DOI:** 10.3390/brainsci11060782

**Published:** 2021-06-13

**Authors:** Daniela Theodoridou, Pavlos Christodoulides, Victoria Zakopoulou, Maria Syrrou

**Affiliations:** 1 Laboratory of Biology, Faculty of Medicine, School of Health Sciences, University of Ioannina, 45110 Ioannina, Greece; danielatheodoridou@gmail.com; 2 Laboratory of Physiology, Faculty of Medicine, School of Health Sciences, University of Ioannina, 45110 Ioannina, Greece; pchristo@uoi.gr; 3 Department of Speech and Language Therapy, School of Health Sciences, University of Ioannina, 45110 Ioannina, Greece; vzakop@uoi.gr

**Keywords:** developmental dyslexia, early life stress, HPA axis, stress-associated endophenotype

## Abstract

Developmental dyslexia (DD) is a multifactorial, specific learning disorder. Susceptibility genes have been identified, but there is growing evidence that environmental factors, and especially stress, may act as triggering factors that determine an individual’s risk of developing DD. In DD, as in most complex phenotypes, the presence of a genetic mutation fails to explain the broad phenotypic spectrum observed. Early life stress has been repeatedly associated with the risk of multifactorial disorders, due to its effects on chromatin regulation, gene expression, HPA axis function and its long-term effects on the systemic stress response. Based on recent evidence, we discuss the potential role of stress on DD occurrence, its putative epigenetic effects on the HPA axis of affected individuals, as well as the necessity of early and appropriate intervention, based on the individual stress-associated (endo)phenotype.

## 1. Introduction

Developmental dyslexia (DD) is a hereditary, multifactorial, specific learning disorder characterized by difficulties to acquire the age-appropriate learning skills (reading, writing, and spelling) [1,2,3]. DD affects 5–12% of individuals, resulting in unfavourable educational and psychosocial outcomes [4,5]. It is mostly diagnosed in school age children, with normal or above average IQ scores, without neurological or sensory conditions [6,7,8]. DD presents phenotypic and genetic heterogeneity and 40–60% heritability, namely a strong genetic component [9]. The individual’s genetic architecture, with hundreds of different variants in interplay with different individual epigenomes, confers the complexity of DD as a heterogeneous disorder. Consequently, many aetiologies may confer to the DD phenotype. As it has been already documented for other neurodevelopmental conditions, phenotypes result from the interaction of risk genes epigenetically regulated by environmental factors, as well as risk genes and/or environmental cues affecting brain connectivity. Variant frequency in a population, interactions between genes and variants, and gene penetrance may further contribute to the heterogeneity of complex phenotypes [10]. Children who develop DD in preschool age have been reported to exhibit emotional (sadness, inadequacy, reduced happiness and self-esteem, anxiety, shyness, suicide) and executive behavior disorders (social isolation, disruption or aggression) in adolescence and adulthood [11]. Despite their comorbidity with dyslexia, such phenotypes do not imply a cause-effect mechanism for DD occurrence, but rather a putative implication of its maintenance [12,13]. In the era of advanced technologies (imaging) and omics, research on DD unravels much more complex aetiologies than those originally supported [14]. Such genome wide approaches have only started to deepen our understanding on DD phenotypes, not by rejecting the established knowledge but by adding information on complex molecular mechanisms and their interplay with risk genes [15,16]. DD might arise from stress response system dysregulation, due to excessive stress exposure [5,15,17]. The interaction between neural, genetic, cognitive and environmental factors is believed to govern susceptibility, and its broad phenotypic spectrum may be due to the individual’s genomic architecture, plus the epigenetic alterations [18]. Some combinations of the aforementioned factors have been proposed to increase and others to decrease DD liability, explaining its heritability variation [5,18,19].

## 2. Why Gene-Only DD Models Lack Cause-Effect Specificity?

DD candidate gene-association and GWAS studies have identified susceptibility genes and genetic risk variants [2,14,20,21]. The absence of cause-effect mutations suggests that the presence of a genetic alteration may not sufficiently explain the phenotype or provide a solid clue to etiopathology [21]. The presence of a mutation does not confer susceptibility to DD per se, but depends on the environmental trigger that may change penetrance and/or expressivity, reflecting the complex etiopathogenesis. Thus, DD is rather the result of the combinatory effects of environmental triggers and risk genes. Interactions between genes and environment can modulate genetic susceptibility to DD-related phenotypes [22,23,24,25]. Specifically, gene-gene (GxG/epistasis) and genotype-environment (GxE) interactions could modify the expression of individual genes, further influencing genetic susceptibility and broadening the DD phenotypic spectrum. The observed phenotypic heterogeneity of DD, including its spectrum of phenotypes and endophenotypes, may be further explained by inter-individual variation and genotype-epigenotype interactions [26,27,28]. Table 1 summarizes literature findings that advocate the role of environment in the putative modulation of genetic susceptibility, thus affecting the individual DD risk.

## 3. A Role for Stress in the DD Phenotype

A plethora of models have been proposed for DD etiopathology, supporting the critical role of the environment [15,26]. Environmental triggers, such as stress, maternal diet, and lifestyle have been proposed as modulators of genetic susceptibility via posttranslational, epigenetic interactions [23,28,29,30,31,32,33,34,35,36,37,38,39,40]. Stress itself seems to be a fundamental environmental factor that could lead to DD or influence DD-related phenotypes, irrespective of the presence or absence of genetic mutations in key risk genes. In individuals with genetic mutations in dyslexia risk genes, stress may act epigenetically, and affect brain plasticity, leading to more or less profound phenotypes. It is reasonable to assume that interindividual genetic and epigenetic variability could explain inter- or intra-familial differences. Anatomical dysfunctions mainly located in the left hemisphere (temporoparietal, occipitotemporal regions and frontal gyrus) have been observed in individuals with DD [6,41,42,43,44]. A recently proposed model for neurodevelopmental disorders associates the hemispheric asymmetries, and therefore the observed atypical lateralization, with stress (chronic and early life stress, ELS) and neurodevelopment [45]. It was proposed that the timing and intensity of the stressor could result in hemispheric asymmetries and more or less prominent phenotypes [45,46]. This model could apply and explain at least the stress related DD endophenotype. Stress, and especially ELS during critical neurodevelopmental periods, is known to influence neuroplasticity [47]. The influence of stress on the two hemispheres is possibly not the same, as the two hemispheres present differences as far as it concerns their regulatory impact on the hypothalamic-pituitary-adrenal (HPA) axis [45,48]. Thus, brain asymmetries, like the ones observed in DD, if seen under the prism of early life recurrent stress exposure, could be the result of the epigenetic effects on the developing brain structures and neuroplasticity itself. Therefore, a DD putative endophenotype could be solely due to stress [49]. In such cases, an enriched or supportive environment may counteract early life adversities, presenting favourable outcomes after early intervention [50,51].

## 4. Stress in Pathophysiology and Behavior

The neuroendocrine response to stress is mediated via the HPA axis and maintains the systemic homeostasis. The cascade of interactions among HPA glands commences soon after a stressful experience. HPA axis dysregulations lead to unsuccessful stress-coping mechanisms and have been associated with many different pathologies [52]. It is well established that early life stressful experiences are associated with the development of psychopathology, due to their effects on early programming and on the function of the HPA axis [53,54,55,56,57]. ELS has also been associated with learning difficulties and neurodevelopmental disorders [15,58,59,60]. Stress is a core concept in the current perspective of recent DD approaches. In this context, children with DD, who experience chronic stress due to constant fear, anticipation of failure, frustration, low self-esteem and loneliness in school environments react with a positive adaptation [57,59]. The earlier in life and prolonged in duration the feelings of failure that children with DD experience, the more their ability to acquire specific learning or academic skills is affected [2,61,62]. These feelings potentially lead to frustration, lack of motivation, negative self-esteem, aggressiveness and vulnerability [63,64]. Likewise, experiences of learned helplessness in children with DD are predictive of internalizing and externalizing problems [65]. It is reported that children ‘at risk’ of DD face early difficulties in the school environment including language, cognitive and motor functions, and impaired socio-emotional skills [66,67]. Apparently, children with such traits fail to cope with frustration and stress [68,69]. It was suggested that a dysregulation of the HPA axis may play the critical role in dyslexia, reflecting long-term adaptation and adjustments to the “threatening” learning environment [57,70,71]. The evidence that psychosocial support may act positively on the HPA axis function, either modifying it or preventing possible alterations, agrees with the dynamic nature of epigenetic mechanisms and necessitates early intervention [72]. The adaptive response of an organism when exposed to a stressful environmental condition represents an integral part of physiologic homeostasis and includes both behavioral and neuroendocrine adjustments [73,74]. Stress exposure activates the HPA axis, which is essential for the neuroendocrine maintenance of homeostasis [75,76,77]. Alterations in neurotransmission and synaptic plasticity were documented in HPA axis-associated brain regions (frontal cortex, hippocampus, and amygdala), in cases of chronic ELS [78,79,80]. Such regions, that mediate decision making in the context of the fight-flight response, are also targets of stress hormones [81]. Initially, deficits and abnormality in neuronal migration in the prefrontal cortex and amygdala were proposed as causal factors for DD etiopathology. However, this opinion has recently been questioned [16,26,60,82]. Growing evidence supports the role of epigenetic effects of early acute or chronic stress (prenatal, perinatal and/or adult social stress) on neuroendocrine effectiveness, social behavior and cognitive ability [27,43,83,84,85]. This new research approach could fill the gap in understanding DD etiopathogenesis, paving the way for appropriate intervention. Environmental and psychosocial stress during critical developmental periods could modulate gene expression via epigenetic modifications, as has been observed in neurodevelopmental disorders and putatively in learning deficits and dyslexia [5,58,81,83,86]. Neuroplasticity has been considered as highly sensitive to ELS [87]. Recent research has demonstrated the epigenetic effect of stress and its dynamic, reversible nature in a rodent model of early life adversity (maternal deprivation). As observed, the levels of a neurotrophic factor implicated in learning, memory and neuroplasticity (brain-derived neurotrophic factor, BDNF) appeared reduced. However, exposure to an enriched environment has the potential to alter the BDNF molecular pattern and putatively affect neurogenesis [49,88,89,90,91]. Recent findings associated dyslexia with stress-related genes, changes in the HPA axis and neuroplasticity. The stressful learning process during childhood and later life can epigenetically influence neuroplasticity, altering the expression profiles of the HPA axis- and neuroplasticity-related genes, as well as influencing personality characteristics [15,49,57,92].

## 5. Is It Time to Include Epigenetics in the Diagnosis of Complex Phenotypes?

Individuals with the same genotype may respond differently to alternate environmental cues, and this GxE interplay may give rise to different phenotypes deriving from the same genotype. Epigenetic mechanisms, such as the well-studied methylation, histone modifications and non-coding regulatory RNAs (miRNA, lncRNAs), are involved in epigenetic programming [93,94]. Exposure to environmental stressors during critical stages of fetal development or during neonatal or early childhood might be a predisposing factor for disease later in life, and epigenetic mechanisms are proposed to be the mediators [95]. DD individual risk is influenced by the developmental stage of the stress exposure, the intensity and duration of the stressor, regardless of the presence of genetic mutations. DD risk genes may be stress-regulated or not. Stress-regulated DD risk genes might be especially prone to contribute to a DD endophenotype. In the case of DD individuals with such risk genes, an interaction of the genetic background with environmental stressors (represented by epigenetic tags) affects neuroplasticity (GxE interaction model) [25]. Early intervention could be especially beneficial to such an endophenotype, regarding the dynamic and reversible nature of epigenetic modifications. The presence of a stress-only induced and epigenetically regulated DD endophenotype is in agreement with the “context sensitivity hypothesis” [57,96,97]. According to this hypothesis, children with dyslexia develop an adaptive response to stress, namely “a careless” attitude [57,96]. Constant stress exposure and chronic exposure to glucocorticoids results in altered circulating cortisol levels and HPA axis hyperfunction [57,96]. This contributes to cortisol resistance, leading to the adaptation and lowering of baseline reactions to stress due to the recurrent expectation of stressful learning difficulties. This chronic exposure to glucocorticoids affects brain structure development and could explain this “careless” behavioral outcome [49,57,97,98,99].

## 6. Conclusions

Complex, heterogeneous, environment-dependent phenotypes require a multidisciplinary approach to investigation. Neuroendocrine, physiological, genetic and epigenetic data collection and analysis is important for a better understanding of the multiple DD aetiologies and the application of the most effective intervention method [25,49]. Only a few studies about DD have so far examined parameters regarding environmental conditions (preconceptual, in utero and postnatal), familial acceptance and milieu, socioeconomic status of the family, lifestyle and upbringing conditions [25]. It is time to elucidate and include the role of stress in DD diagnosis, while assessing both the aforementioned factors and psychosocial stressors (e.g., peer-acceptance, bullying in the school environment). Such information may help identify phenotype-specific, stress-related biomarkers, and pave the way to a multifaceted diagnostic procedure. An in-depth study and understanding of biological and psychosocial factors will establish a new approach on early diagnosis and intervention procedures, positively influencing the life of children with DD [49,57,88].

## Figures and Tables

**Table 1:** Environmental components that may influence developmental dyslexia and sources of evidence.

Environmental Component	References
Education	The interface between genetics and psychology: lessons from developmental dyslexia [28]
Family environment, parental education, living environment	Personality, Behavior Characteristics, and Life Quality Impact of Children with Dyslexia [29]
Education of the mother, family history of language or psychiatric problems, perinatal problems and health problems in early childhood	Environmental and genetic variables related with alterations in language acquisition in early childhood [30]
Maternal smoking, family education, birth weight, socioeconomic status	Genetic and environmental risk factors for developmental dyslexia in children: systematic review of the last decade [31]
Socioeconomic status	Socioeconomic status and cognitive functioning: moving from correlation to causation [32] Association of Child Poverty, Brain Development and Academic Achievement [33]
Socioeconomic status, home literacy environment, family stresses, and child health	Child and environmental risk factors predicting readiness for learning in children at high risk of dyslexia [34]

## References

[1] Schumacher J., Hoffmann P., Schmal C., Schulte-Korne G., Nothen M.M. (2007). Genetics of dyslexia: The evolving landscape. J. Med. Genet..

[2] Mascheretti S., De Luca A., Trezzi V., Peruzzo D., Nordio A., Marino C., Arrigoni F. (2017). Neurogenetics of developmental dyslexia: From genes to behavior through brain neuroimaging and cognitive and sensorial mechanisms. Transl. Psychiatry.

[3] (2013). Diagnostic and Statistical Manual of Mental Disorders.

[4] Undheim A.M. (2003). Dyslexia and psychosocial factors. A follow-up study of young Norwegian adults with a history of dyslexia in childhood. Nord. J. Psychiatry.

[5] Zuk J., Dunstan J., Norton E., Yu X., Ozernov-Palchik O., Wang Y., Hogan T.P., Gabrieli J.D.E., Gaab N. (2021). Multifactorial pathways facilitate resilience among kindergarteners at risk for dyslexia: A longitudinal behavioral and neuroimaging study. Dev. Sci..

[6] Peterson R.L., Pennington B.F. (2015). Developmental dyslexia. Annu. Rev. Clin. Psychol..

[7] Tanaka H., Black J.M., Hulme C., Stanley L.M., Kesler S.R., Whitfield-Gabrieli S., Reiss A.L., Gabrieli J.D., Hoeft F. (2011). The brain basis of the phonological deficit in dyslexia is independent of IQ. Psychol. Sci..

[8] Protopapas A., Parrila R. (2019). Dyslexia: Still Not a Neurodevelopmental Disorder. Brain Sci..

[9] Plomin R., Kovas Y. (2005). Generalist genes and learning disabilities. Psychol. Bull..

[10] Daskalakis N.P., Bagot R.C., Parker K.J., Vinkers C.H., de Kloet E.R. (2013). The three-hit concept of vulnerability and resilience: Toward understanding adaptation to early-life adversity outcome. Psychoneuroendocrinology.

[11] Undheim A.M., Wichstrøm L., Sund A.M. (2011). Emotional and Behavioral Problems Among School Adolescents With and Without Reading Difficulties as Measured by the Youth Self-Report: A One-Year Follow-Up Study. Scand. J. Educ. Res..

[12] Terras M.M., Thompson L.C., Minnis H. (2009). Dyslexia and psycho-social functioning: An exploratory study of the role of self-esteem and understanding. Dyslexia.

[13] Zakopoulou V., Mavreas V., Christodoulides P., Lavidas A., Fili E., Georgiou G., Dimakopoulos G., Vergou M. (2014). Specific learning difficulties: A retrospective study of their co morbidity and continuity as early indicators of mental disorders. Res. Dev. Disabil..

[14] Gialluisi A., Andlauer T.F., Mirza-Schreiber N., Moll K., Becker J., Hoffmann P., Ludwig K.U., Czamara D., St Pourcain B., Honbolygó F. (2020). Genome-wide association study reveals new insights into the heritability and genetic correlates of developmental dyslexia. Mol. Psychiatry.

[15] Kershner J.R. (2020). An Evolutionary Perspective of Dyslexia, Stress, and Brain Network Homeostasis. Front. Hum. Neurosci..

[16] Guidi L.G., Velayos-Baeza A., Martinez-Garay I., Monaco A.P., Paracchini S., Bishop D.V.M., Molnar Z. (2018). The neuronal migration hypothesis of dyslexia: A critical evaluation 30 years on. Eur. J. Neurosci..

[17] Peters L., Ansari D. (2019). Are specific learning disorders truly specific, and are they disorders?. Trends Neurosci. Educ..

[18] van Bergen E., van der Leij A., de Jong P.F. (2014). The intergenerational multiple deficit model and the case of dyslexia. Front. Hum. Neurosci..

[19] Pennington B.F. (2006). From single to multiple deficit models of developmental disorders. Cognition.

[20] Gialluisi A., Andlauer T.F.M., Mirza-Schreiber N., Moll K., Becker J., Hoffmann P., Ludwig K.U., Czamara D., St Pourcain B., Brandler W. (2019). Genome-wide association scan identifies new variants associated with a cognitive predictor of dyslexia. Transl. Psychiatry.

[21] Smith S.D. (2011). Approach to epigenetic analysis in language disorders. J. Neurodev. Disord..

[22] Cho K., Frijters J.C., Zhang H., Miller L.L., Gruen J.R. (2013). Prenatal exposure to nicotine and impaired reading performance. J. Pediatr..

[23] Romeo R.R., Christodoulou J.A., Halverson K.K., Murtagh J., Cyr A.B., Schimmel C., Chang P., Hook P.E., Gabrieli J.D.E. (2018). Socioeconomic Status and Reading Disability: Neuroanatomy and Plasticity in Response to Intervention. Cereb. Cortex.

[24] Ozernov-Palchik O., Norton E.S., Wang Y., Beach S.D., Zuk J., Wolf M., Gabrieli J.D.E., Gaab N. (2019). The relationship between socioeconomic status and white matter microstructure in pre-reading children: A longitudinal investigation. Hum. Brain Mapp..

[25] Mascheretti S., Bureau A., Battaglia M., Simone D., Quadrelli E., Croteau J., Cellino M.R., Giorda R., Beri S., Maziade M. (2013). An assessment of gene-by-environment interactions in developmental dyslexia-related phenotypes. Genes Brain Behav..

[26] Kershner J.R. (2019). Neurobiological systems in dyslexia. Trends Neurosci. Educ..

[27] D’Souza S., Backhouse-Smith A., Thompson J.M., Slykerman R., Marlow G., Wall C., Murphy R., Ferguson L.R., Mitchell E.A., Waldie K.E. (2016). Associations Between the KIAA0319 Dyslexia Susceptibility Gene Variants, Antenatal Maternal Stress, and Reading Ability in a Longitudinal Birth Cohort. Dyslexia.

[28] Bishop D.V. (2015). The interface between genetics and psychology: Lessons from developmental dyslexia. Proc. R. Soc. B Biol. Sci..

[29] Huang Y., He M., Li A., Lin Y., Zhang X., Wu K. (2020). Personality, Behavior Characteristics, and Life Quality Impact of Children with Dyslexia. Int J Environ Res Public Health.

[30] Moriano-Gutierrez A., Colomer-Revuelta J., Sanjuan J., Carot-Sierra J.M. (2017). [Environmental and genetic variables related with alterations in language acquisition in early childhood]. Rev Neurol.

[31] Becker N., Vasconcelos M., Oliveira V., Santos F.C.D., Bizarro L., Almeida R.M.M., Salles J.F., Carvalho M.R.S. (2017). Genetic and environmental risk factors for developmental dyslexia in children: Systematic review of the last decade. Dev Neuropsychol.

[32] Duncan G.J., Magnuson K. (2012). Socioeconomic status and cognitive functioning: moving from correlation to causation. Wiley Interdiscip Rev Cogn Sci.

[33] Hair N.L., Hanson J.L., Wolfe B.L., Pollak S.D. (2015). Association of Child Poverty, Brain Development, and Academic Achievement. JAMA Pediatr.

[34] Dilnot J., Hamilton L., Maughan B., Snowling M.J. (2017). Child and environmental risk factors predicting readiness for learning in children at high risk of dyslexia. Dev Psychopathol.

[35] Grigorenko E.L. (2001). Developmental dyslexia: An update on genes, brains, and environments. J. Child. Psychol. Psychiatry.

[36] Hay I., Hynes K.L., Burgess J.R. (2019). Mild-to-Moderate Gestational Iodine Deficiency Processing Disorder. Nutrients.

[37] Rash B.G., Micali N., Huttner A.J., Morozov Y.M., Horvath T.L., Rakic P. (2018). Metabolic regulation and glucose sensitivity of cortical radial glial cells. Proc. Natl. Acad. Sci. USA.

[38] Walsh K., McCormack C.A., Webster R., Pinto A., Lee S., Feng T., Krakovsky H.S., O’Grady S.M., Tycko B., Champagne F.A. (2019). Maternal prenatal stress phenotypes associate with fetal neurodevelopment and birth outcomes. Proc. Natl. Acad. Sci. USA.

[39] Pennington B.F., Bishop D.V. (2009). Relations among speech, language, and reading disorders. Annu. Rev. Psychol..

[40] Becker S.P., Leopold D.R., Burns G.L., Jarrett M.A., Langberg J.M., Marshall S.A., McBurnett K., Waschbusch D.A., Willcutt E.G. (2016). The Internal, External, and Diagnostic Validity of Sluggish Cognitive Tempo: A Meta-Analysis and Critical Review. J. Am. Acad. Child. Adolesc. Psychiatry.

[41] Galaburda A.M. (1999). Developmental dyslexia: A multilevel syndrome. Dyslexia.

[42] Peterson R.L., Pennington B.F. (2012). Developmental dyslexia. Lancet.

[43] Raskind W., Peter B., Richards T., Eckert M., Berninger V. (2013). The Genetics of Reading Disabilities: From Phenotypes to Candidate Genes. Front. Psychol..

[44] Mascheretti S., Bureau A., Trezzi V., Giorda R., Marino C. (2015). An assessment of gene-by-gene interactions as a tool to unfold missing heritability in dyslexia. Hum. Genet..

[45] Berretz G., Wolf O.T., Gunturkun O., Ocklenburg S. (2020). Atypical lateralization in neurodevelopmental and psychiatric disorders: What is the role of stress?. Cortex.

[46] O’Donnell K.J., Meaney M.J. (2017). Fetal Origins of Mental Health: The Developmental Origins of Health and Disease Hypothesis. Am. J. Psychiatry.

[47] Burga A., Lehner B. (2012). Beyond genotype to phenotype: Why the phenotype of an individual cannot always be predicted from their genome sequence and the environment that they experience. FEBS J..

[48] Mai S., Braun J., Probst V., Kammer T., Pollatos O. (2019). Changes in emotional processing following interoceptive network stimulation with rTMS. Neuroscience.

[49] Zakopoulou V., Vlaikou A.M., Darsinou M., Papadopoulou Z., Theodoridou D., Papageorgiou K., Alexiou G.A., Bougias H., Siafaka V., Zoccolotti P. (2019). Linking Early Life Hypothalamic-Pituitary-Adrenal Axis Functioning, Brain Asymmetries, and Personality Traits in Dyslexia: An Informative Case Study. Front. Hum. Neurosci..

[50] Schmitz J., Gunturkun O., Ocklenburg S. (2019). Building an Asymmetrical Brain: The Molecular Perspective. Front. Psychol..

[51] Elbau I.G., Cruceanu C., Binder E.B. (2019). Genetics of Resilience: Gene-by-Environment Interaction Studies as a Tool to Dissect Mechanisms of Resilience. Biol. Psychiatry.

[52] DeMorrow S. (2018). Role of the Hypothalamic-Pituitary-Adrenal Axis in Health and Disease. Int. J. Mol. Sci..

[53] Papadopoulou Z., Vlaikou A.M., Theodoridou D., Komini C., Chalkiadaki G., Vafeiadi M., Margetaki K., Trangas T., Turck C.W., Syrrou M. (2019). Unraveling the Serum Metabolomic Profile of Post-partum Depression. Front. Neurosci..

[54] Papadopoulou Z., Vlaikou A.M., Theodoridou D., Markopoulos G.S., Tsoni K., Agakidou E., Drosou-Agakidou V., Turck C.W., Filiou M.D., Syrrou M. (2019). Stressful Newborn Memories: Pre-Conceptual, In Utero, and Postnatal Events. Front. Psychiatry.

[55] McEwen B.S., Gianaros P.J. (2011). Stress- and allostasis-induced brain plasticity. Annu. Rev. Med..

[56] McGowan P.O., Matthews S.G. (2018). Prenatal Stress, Glucocorticoids, and Developmental Programming of the Stress Response. Endocrinology.

[57] Espin L., Garcia I., Del Pino Sanchez M., Roman F., Salvador A. (2019). Effects of psychosocial stress on the hormonal and affective response in children with dyslexia. Trends Neurosci. Educ..

[58] Gomes F.V., Zhu X., Grace A.A. (2019). Stress during critical periods of development and risk for schizophrenia. Schizophr. Res..

[59] Bale T.L. (2011). Sex differences in prenatal epigenetic programming of stress pathways. Stress.

[60] Kershner J.R. (2020). Dyslexia as an adaptation to cortico-limbic stress system reactivity. Neurobiol. Stress.

[61] Haft S.L., Myers C.A., Hoeft F. (2016). Socio-Emotional and Cognitive Resilience in Children with Reading Disabilities. Curr. Opin. Behav. Sci..

[62] Zoccolotti P., de Jong P.F., Spinelli D. (2016). Editorial: Understanding Developmental Dyslexia: Linking Perceptual and Cognitive Deficits to Reading Processes. Front. Hum. Neurosci..

[63] Tarabia E., Abu-Rabia S. (2016). Social Competency, Sense of Loneliness and Self-Image among Reading Disabled (RD) Arab Adolescents. Creat. Educ..

[64] Zakopoulou V., Pashou T., Tzavelas P., Christodoulides P., Anna M., Iliana K. (2013). Learning difficulties: A retrospective study of their co morbidity and continuity as indicators of adult criminal behaviour in 18-70-year-old prisoners. Res. Dev. Disabil..

[65] Sorrenti L., Spadaro L., Mafodda A.V., Scopelliti G., Orecchio S., Filippello P. (2019). The predicting role of school Learned helplessness in internalizing and externalizing problems. An exploratory study in students with Specific Learning Disorder. Mediterr. J. Clin. Psychol..

[66] Rouse H.L., Fantuzzo J.W. (2006). Validity of the Dynamic Indicators for Basic Early Literacy Skills as an indicator of early literacy for urban kindergarten children. School Psychol. Rev..

[67] Oehler-Stinnett J., Boykin C. (2001). Convergent, discriminant, and predictive validity of the Teacher Rating of Academic Achievement Motivation (TRAAM) with the ACTeRs-TF and the BASC-TRS. J. Psychoeduc. Assess..

[68] Duncan G.J., Dowsett C.J., Claessens A., Magnuson K., Huston A.C., Klebanov P., Pagani L.S., Feinstein L., Engel M., Brooks-Gunn J. (2007). School readiness and later achievement. Dev. Psychol..

[69] Dobbs J., Doctoroff G.L., Fisher P.H., Arnold D.H. (2006). The association between preschool children’s socio-emotional functioning and their mathematical skills. J. Appl. Dev. Psychol..

[70] Alexander-Passe N. (2008). The sources and manifestations of stress amongst school-aged dyslexics, compared with sibling controls. Dyslexia.

[71] Taj R.A., Malik M. (2010). Conclusive study to uncover the attributors for success and failure of learning disabled children. Eur. J. Soc. Sci..

[72] Gunnar M.R., Talge N.M., Herrera A. (2009). Stressor paradigms in developmental studies: What does and does not work to produce mean increases in salivary cortisol. Psychoneuroendocrinology.

[73] Agorastos A., Nicolaides N.C., Bozikas V.P., Chrousos G.P., Pervanidou P. (2019). Multilevel Interactions of Stress and Circadian System: Implications for Traumatic Stress. Front. Psychiatry.

[74] Tsigos C., Kyrou I., Kassi E., Chrousos G.P., Feingold K.R., Anawalt B., Boyce A., Chrousos G., de Herder W.W., Dungan K., Grossman A., Hershman J.M., Hofland J., Kaltsas G. (2000). Stress: Endocrine Physiology and Pathophysiology. Endotext.

[75] Tsigos C., Chrousos G.P. (2002). Hypothalamic-pituitary-adrenal axis, neuroendocrine factors and stress. J. Psychosom. Res..

[76] Gunnar M.R., Donzella B. (2002). Social regulation of the cortisol levels in early human development. Psychoneuroendocrinology.

[77] Hennessy J.W. (1979). Stress, Arousal, and the Pituitary-Adrenal System: A Psychoendocrine Hypothesis. pascal-francis.inist.fr/vibad/index.php?action=getRecordDetail&idt=PASCAL8050152111.

[78] Gardner K.L., Hale M.W., Lightman S.L., Plotsky P.M., Lowry C.A. (2009). Adverse early life experience and social stress during adulthood interact to increase serotonin transporter mRNA expression. Brain Res..

[79] Vogel S., Schwabe L. (2016). Learning and memory under stress: Implications for the classroom. NPJ Sci. Learn..

[80] van Bodegom M., Homberg J.R., Henckens M. (2017). Modulation of the Hypothalamic-Pituitary-Adrenal Axis by Early Life Stress Exposure. Front. Cell Neurosci..

[81] FeldmanHall O., Glimcher P., Baker A.L., Collaboration N.P., Phelps E.A. (2019). The Functional Roles of the Amygdala and Prefrontal Cortex in Processing Uncertainty. J. Cogn. Neurosci..

[82] Jiménez-Bravo M., Marrero V., Benítez-Burraco A. (2017). An oscillopathic approach to developmental dyslexia: From genes to speech processing. Behav. Brain Res..

[83] Lamb Y.N., Thompson J.M., Murphy R., Wall C., Kirk I.J., Morgan A.R., Ferguson L.R., Mitchell E.A., Waldie K.E., ABC Study Group (2014). Perceived stress during pregnancy and the catechol-O-methyltransferase (COMT) rs165599 polymorphism impacts on childhood IQ. Cognition.

[84] Elzinga B.M., Roelofs K. (2005). Cortisol-induced impairments of working memory require acute sympathetic activation. Behav. Neurosci..

[85] Roozendaal B., McEwen B.S., Chattarji S. (2009). Stress, memory and the amygdala. Nat. Rev. Neurosci..

[86] Gudsnuk K., Champagne F.A. (2012). Epigenetic influence of stress and the social environment. ILAR J..

[87] McEwen B.S., Nasca C., Gray J.D. (2016). Stress Effects on Neuronal Structure: Hippocampus, Amygdala, and Prefrontal Cortex. Neuropsychopharmacology.

[88] Huang Y., Xu C., He M., Huang W., Wu K. (2020). Saliva cortisol, melatonin levels and circadian rhythm alterations in Chinese primary school children with dyslexia. Medicine (Baltimore).

[89] Menezes J., Souto das Neves B.H., Goncalves R., Benetti F., Mello-Carpes P.B. (2020). Maternal deprivation impairs memory and cognitive flexibility, effect that is avoided by environmental enrichment. Behav. Brain Res..

[90] Cowansage K.K., LeDoux J.E., Monfils M.H. (2010). Brain-derived neurotrophic factor: A dynamic gatekeeper of neural plasticity. Curr. Mol. Pharmacol..

[91] Rattiner L.M., Davis M., Ressler K.J. (2005). Brain-derived neurotrophic factor in amygdala-dependent learning. Neuroscientist.

[92] Karten Y.J., Olariu A., Cameron H.A. (2005). Stress in early life inhibits neurogenesis in adulthood. Trends Neurosci..

[93] Cavalli G., Heard E. (2019). Advances in epigenetics link genetics to the environment and disease. Nature.

[94] Zhang L., Lu Q., Chang C. (2020). Epigenetics in Health and Disease. Adv. Exp. Med. Biol..

[95] Heindel J.J., Vandenberg L.N. (2015). Developmental origins of health and disease: A paradigm for understanding disease cause and prevention. Curr. Opin. Pediatr..

[96] Thomson M.E. (1990). Developmental Dyslexia: Studies in Disorders of Communication.

[97] Boyce W.T., Ellis B.J. (2005). Biological sensitivity to context: I. An evolutionary-developmental theory of the origins and functions of stress reactivity. Dev. Psychopathol..

[98] Carrion V.G., Weems C.F., Reiss A.L. (2007). Stress predicts brain changes in children: A pilot longitudinal study on youth stress, posttraumatic stress disorder, and the hippocampus. Pediatrics.

[99] De Bellis M.D., Kuchibhatla M. (2006). Cerebellar volumes in pediatric maltreatment-related posttraumatic stress disorder. Biol. Psychiatry.

